# Functional characterization of a novel aminoglycoside phosphotransferase, APH(9)-Ic, and its variant from *Stenotrophomonas maltophilia*

**DOI:** 10.3389/fcimb.2022.1097561

**Published:** 2023-01-09

**Authors:** Weina Shi, Junwan Lu, Chunlin Feng, Mengdi Gao, Anqi Li, Shuang Liu, Lei Zhang, Xueya Zhang, Qiaoling Li, Hailong Lin, Xi Lin, Kewei Li, Hailin Zhang, Yunliang Hu, Guangli Wang, Qiyu Bao, Weiyan Jiang

**Affiliations:** ^1^ The Second Affiliated Hospital and Yuying Children’s Hospital, Wenzhou Medical University, Wenzhou, China; ^2^ Key Laboratory of Medical Genetics of Zhejiang Province, Key Laboratory of Laboratory Medicine, Ministry of Education, China, School of Laboratory Medicine and Life Sciences, Wenzhou Medical University, Wenzhou, China; ^3^ Medical Molecular Biology Laboratory, School of Medicine, Jinhua Polytechnic, Jinhua, China; ^4^ School of Medicine and Health, Lishui University, Lishui, China

**Keywords:** APH(9)-Ic, aminoglycoside resistance, aminoglycoside-modifying enzyme, aminoglycoside 9-phosphotransferase, *Stenotrophomonas maltophilia*

## Abstract

**Background:**

The intrinsic resistance mechanism plays an essential role in the bacterial resistance to a variety of the antimicrobials. The aim of this study is to find the chromosome-encoded novel antimicrobial resistance gene in the clinical isolate.

**Methods:**

The function of the predicted resistance gene was verified by gene cloning and antibiotic susceptibility test. Recombinant protein expression and enzyme kinetic studies were performed to explore the in vivo activity of the enzyme. Expression of the resistance gene exposed to antimicrobial was determined by RT-qPCR. Whole genome sequencing and bioinformatic analysis were applied to analyze the genetic context of the resistance gene.

**Results:**

The novel aminoglycoside (AG) resistance genes designated aph(9)-Ic and aph(9)-Ic1 confer resistance to spectinomycin, and a recombinant strain harboring aph(9)-Ic (pMD19-T-aph(9)-Ic/DH5α) showed a significantly increased minimum inhibitory concentration (MIC) level against spectinomycin compared with the control strains (DH5α and pMD19-T/DH5α). The result of the kinetic analysis of APH(9)-Ic was consistent with the MIC result for the recombinant pMD19-T-aph(9)-Ic/DH5α, showing the efficient catalytic activity for spectinomycin [kcat/Km ratio = (5.58 ± 0.31) × 104 M−1·s−1]. Whole-genome sequencing demonstrated that the aph(9)-Ic gene was located on the chromosome with a relatively conserved genetic environment, and no mobile genetic element was found in its surrounding region. Among all the function-characterized resistance genes, APH(9)-Ic shares the highest amino acid sequence identity of 33.75% with APH(9)-Ia.

**Conclusion:**

We characterized a novel AG resistance gene aph(9)-Ic and its variant aph(9)-Ic1 that mediated spectinomycin resistance from S. maltophilia. The identification of the novel AG resistance genes will assist us in elucidating the complexity of resistance mechanisms in microbial populations.

## Introduction

Aminoglycosides (AGs) play an important role in bacterial warfare. Some of them are the secondary metabolites of certain bacteria (Kudo and Eguchi, 2016). Due to the advent of other broad-spectrum antibiotics with fewer side effects and the emergence of AG-resistant strains, the use of AGs to treat infectious diseases has become more cautious in clinical practice (Becker and Cooper, 2013; Kudo and Eguchi, 2016). Currently, with the dramatically increasing rate of nosocomial infections caused by bacteria that have become increasingly resistant to both β-lactam antibiotics and fluoroquinolones, focus has returned to AGs as one of the few remaining treatment options, particularly for carbapenem-resistant Gram-negative pathogens (Dozzo and Moser, 2010; Becker and Cooper, 2013).

AGs kill bacteria by binding to the bacterial ribosome and inhibiting normal protein synthesis. Specifically, they bind tightly to the highly conserved A-site (the transfer RNA acceptor site) of bacterial 16S ribosomal RNA of the 30S ribosomal subunit (Magnet and Blanchard, 2005). The mode of action of AG antibiotics has been extensively studied and is well understood (Becker and Cooper, 2013). Bacterial resistance to AGs is driven by three major mechanisms: AG-modifying enzymes (AMEs) that inactivate AGs, efflux, and target modification (methylases) (Ramirez and Tolmasky, 2010). The most prevalent form of AG resistance seen in clinical usage is caused by AMEs, which include *O*-phosphotransferases (APHs), *O*-adenyltransferases (ANTs) and *N*-acetyltransferases (AACs) (Wright, 1999; Dozzo and Moser, 2010). To date, the APH(9) class consists of APH(9)-Ia and APH(9)-Ib (https://card.mcmaster.ca/ontology/36292). APH(9) enzymes are the only type of APH capable of inactivating spectinomycin, and they exclusively phosphorylate the hydroxyl group at position 9 (Fong et al., 2010).

*Stenotrophomonas maltophilia* is an aerobic, nonfermentative and Gram-negative bacillus (An and Berg, 2018). In the clinic, a *S. maltophilia* strain of environmental origin has emerged as an important opportunistic pathogen that causes an increasing number of infections among immunocompromised hosts (Chang et al., 2015). Despite its mild virulence, its ability to colonize respiratory tract epithelial cells and the surfaces of medical devices makes it a causative agent of nosocomial diseases (Brooke, 2012; An and Berg, 2018; Gil-Gil et al., 2020). It has been reported that both environmental and clinical isolates of *S. maltophilia* exhibit resistance to multiple antibiotics, including trimethoprim-sulfamethoxazole, aminoglycosides, macrolides, cephalosporins, carbapenems, chloramphenicol, tetracyclines, fluoroquinolones, β-lactam antibiotics, and polymyxins (Brooke, 2012; An and Berg, 2018). The high drug resistance rate can be attributed mainly to the presence of chromosomal genes encoding efflux pumps and antibiotic-inactivating enzymes (Magnet and Blanchard, 2005). Therefore, therapeutic options for the treatment of *S. maltophilia* infections are limited (Looney et al., 2009; Flores-Trevino et al., 2019; Gil-Gil et al., 2020).

In this study, we identified a novel AG phosphotransferase gene of the *aph(9)-I* subfamily designated *aph(9)-Ic* in *S. maltophilia*. Besides the molecular characteristics and enzymatic activity of APH(9)-Ic, the *in vitro* resistance profile of its variant designated *aph(9)-Ic1* was also characterized.

## Materials and methods

### Bacterial strains, plasmids and culture conditions

The *S. maltophilia* strains 142 and 156 carrying the novel *aph(9)-Ic* genes were collected from a tertiary hospital in Wenzhou, Zhejiang, China. The plasmid pMD19-T (Takara Bio, Inc., Dalian, China) was used as the vector for cloning the predicted resistance gene and *Escherichia coli* DH5α was used as the recipient for the recombinant. The pCold I vector was used for cold shock-induced expression of His6-tagged APH(9)-Ic and *E. coli* BL21 was used as the host. The bacterial strains were cultured overnight at 37°C in Luria-Bertani (LB) medium supplemented with the appropriate antimicrobial agents. The strains and plasmids used in this work are listed in **Table 1**.

**Table 1:** Bacteria and plasmids used in this work.

Bacterium or plasmid	Relevant characteristic(s)	Source
Strain		
142	The wild-type strain of *Stenotrophomonas maltophilia* 142	This study
156	The wild-type strain of *Stenotrophomonas maltophilia* 156	This study
DH5**α**	*E. coli* DH5α was used as a host for the cloning of the *aph(9)-Ic* and *aph(9)-Ic1* genes.	Our laboratory collection
BL21	*E. coli* BL21 was used as a host for expression of APH(9)-Ic.	Our laboratory collection
ATCC 25922	*E. coli* ATCC 25922 was used as a quality control for antimicrobial susceptibility testing.	Our laboratory collection
pMD19-T-*aph(9)-Ic*/DH5α	DH5α carrying the recombinant plasmid pMD19-T-*aph(9)-Ic*	This study
pMD19-T-*aph(9)-Ic1*/DH5α	DH5α carrying the recombinant plasmid pMD19-T-*aph(9)-Ic1*	This study
pCold I-*aph(9)-Ic*/BL21	BL21 carrying the recombinant plasmid pCold I-*aph(9)-Ic*	This study
Plasmid		
pMD19-T	Cloning vector for the PCR products of the *aph(9)-Ic* and *aph(9)-Ic1* genes with their upstream promoter regions, ampicillin resistance	Takara Bio, Inc., Dalian, China
pCold I/BL 21	Expression vector for the PCR products of the ORF of the *aph(9)-Ic*, ampicillin resistance	Our laboratory collection

### Genome sequencing, assembly, annotation and bioinformatic analysis

The whole-genomic DNA of *S. maltophilia* 142 and 156 was extracted using an AxyPrep Bacterial Genomic DNA Miniprep Kit (Axygen Biosciences, Union City, CA, USA). Whole-genome sequencing was achieved using the Illumina HiSeq 2500 and PacBio RS II platforms by Shanghai Personal Biotechnology Co., Ltd. (Shanghai, China). Illumina short reads were assembled using SPAdes v3.14.1 (Bankevich et al., 2012), and the average length of short reads was 150 bp. The PacBio long reads of approximately 10 to 20 kb in length were initially assembled using Canu v1.8 software (Koren et al., 2017). The Illumina sequence reads were then mapped onto the primary assembly to correct possible misidentified bases using the Burrows−Wheeler Alignment v0.7.12 tool (Li and Durbin, 2009) and the Genome Analysis Toolkit (Mckenna et al., 2010). The open reading frames (ORFs) were predicted using Prokka v1.14.6 (Seemann, 2014) and further annotated by DIAMOND (Buchfink et al., 2015) against the UniProtKB/Swiss-Prot (http://web.expasy.org/docs/swiss-prot_guideline.html) and NCBI nonredundant (nr) protein databases (https://www.ncbi.nlm.nih.gov/refseq/about/nonredundantproteins/). Annotation of resistance genes was performed using ResFinder (Zankari et al., 2012) and Resistance Gene Identifier (RGI) from the Comprehensive Antibiotic Resistance Database (CARD) (Mcarthur et al., 2013). Multiple sequence alignment and a phylogenetic tree were generated by MEGAX (Kumar et al., 2018) using a maximum parsimony method with 1000 bootstrap replications. The multiple sequence alignment used for generating the phylogenetic tree is displayed in **Supplementary Figure S1**. Analysis of conserved motifs in the APH(9)-Ic sequence was performed using the MEME Suite (http://meme-suite.org/). Gene organization diagrams were drawn with version 2.2.2 of Easyfig (Sullivan et al., 2011). FastANI v1.31 (Jain et al., 2018) was used to calculate the average nucleotide identity (ANI). ProtParam (https://web.expasy.org/protparam/) was used to predict the molecular weight and pI value. CGView Server (Petkau et al., 2010) was used to visualize the basic genomic features of chromosomes and to perform comparative genomic analysis. The promoter region of the *aph(9)-Ic* genes was predicted by BPROM (2016) (http://www.softberry.com/berry.phtml?topic=bprom&group=programs&subgroup=gfindb). The sequence type was analyzed according to multilocus sequence typing (MLST) for *Stenotrophomonas maltophilia* (https://pubmlst.org/bigsdb?db=pubmlst_smaltophilia_seqdef&page=sequenceQuery).

### Antibiotic susceptibility testing

The minimum inhibitory concentrations (MICs) were determined by the agar dilution method with Mueller-Hinton (MH) agar plates. The susceptibility patterns were interpreted according to the Clinical and Laboratory Standards Institute (CLSI, 2022, Performance Standards for Antimicrobial Susceptibility Testing-Twenty-Eighth Edition: M100) guideline breakpoint criteria for *S. maltophilia* and *Pseudomonas aeruginosa*. As no CLSI breakpoint was available, the MIC result for spectinomycin was interpreted according to the publications by Hu et al. (Hu et al., 2017) and Jouybari et al. (Jouybari et al., 2021). *E. coli* ATCC 25922 was included in each test as a quality control standard. The MIC values were determined in triplicate on MH agar plates with twofold serial dilutions of the antimicrobials. The plates were incubated at 37°C for 16-20 hours before the results were analyzed.

### Cloning of the *aph(9)-Ic* gene

Genomic DNA of *S. maltophilia* 142 and 156 was extracted as described above. The DNA fragments carrying the putative resistance genes with their promoter regions were amplified by PCR with the primers listed in **Supplementary Table S1**. Each PCR product was ligated into the pMD19-T vector using a T4 DNA ligase cloning kit (Takara Bio, Inc., Dalian, China). The recombinant plasmids pMD19-T-pro-*aph(9)-Ic* (*S. maltophilia* 142) and pMD19-T-pro-*aph(9)-Ic1* (*S. maltophilia* 156) were transformed into competent *E. coli* DH5α cells by the calcium chloride method, and the transformants were selected on LB agar plates supplemented with 100 μg/mL ampicillin. The cloned insert sequence in the recombinant plasmid of each transformant was further confirmed by both agarose gel electrophoresis and Sanger sequencing of the PCR products (Hangzhou Tsingke Biotechnology Co., Ltd., Hangzhou, China).

### Expression and purification of recombinant APH(9)-Ic

The ORF of the *aph(9)-Ic* gene was amplified with the primers listed in **Supplementary Table S1** and cloned into the pCold I cold shock expression vector. The PCR product and the cloning vector pCold I were digested with both *Bam*HI and *Hind*III (Takara Bio Inc., Dalian, China). Then, the resulting DNA fragments were ligated by T4 DNA ligase (Takara Bio Inc., Dalian, China). The resultant recombinant plasmid pCold I-*aph(9)-Ic* was introduced into *E. coli* BL21 competent cells by the calcium chloride method, and the transformant (pCold I-*aph(9)-Ic*/BL21) was selected on an LB agar plate containing 100 μg/mL ampicillin. The overnight culture of the recombinant strain (pCold I-*aph(9)-Ic*/BL21) was cultured in LB broth containing 100 μg/mL ampicillin at 37°C. When the OD_600_ reached 0.6, isopropyl D-thiogalactopyranoside (IPTG) was added at a concentration of 1 mM to induce the expression of His6-tagged APH(9)-Ic, and cell cultivation was continued for 18 h at 16°C. Cells were harvested by centrifugation (5000 g, 10 min) at 4°C, resuspended in phosphate-buffered saline (pH = 7.4), and disrupted by sonication. The recombinant protein was purified using BeyoGold His-tag Purification Resin, first with nondenaturing wash buffer (50 mM NaH_2_PO_4_, 300 mM NaCl, 2 mM imidazole) and then eluted by the nondenaturing eluent (50 mM NaH_2_PO_4_, 300 mM NaCl, 50 mM imidazole) from the His-tag Protein Purification Kit (Beyotime, Shanghai, China) according to the manufacturer’s instructions (**Supplementary Figure S2**). The His6-tag was removed by enterokinase (GenScript Inc., Nanjing, China) digestion at 4°C for 72 h (**Supplementary Figure S2**). The purity of the protein was confirmed using sodium dodecyl sulfate−polyacrylamide gel electrophoresis (SDS−PAGE) and subsequent staining with Coomassie Brilliant Blue. The protein concentration was determined spectrophotometrically using a BCA protein assay kit (Thermo Fisher Scientific, Rockford, IL, United States).

### Kinetic studies of APH(9)-Ic

The kinetic assay used to monitor APH(9)-Ic activity was performed as reported previously (Lu et al., 2021). Briefly, kinetic parameters for APH(9)-Ic activity were determined by a continuous spectrophotometric assay, which coupled the production of ADP generated upon AG phosphorylation to the oxidation of NADH using the enzymes pyruvate kinase (PK) and lactate dehydrogenase (LD). The rate of ADP production was determined by monitoring the decrease in absorbance at 340 nm using a UV−VIS spectrophotometer (U-3900, Hitachi, Japan) at 37°C. Reactions were initiated by the addition of 250 nM APH(9)-Ic in a 250 μL reaction mixture that contained 100 mM HEPES (pH 7.0), 10 mM MgCl_2_, 20 mM KCl, 2 mM phosphoenolpyruvate (Beijing Solarbio Science & Technology Co., Ltd.), 1 mM NADH (Roche 10107735001), a commercial mixture of PK and LD (Sigma P0294; 18-26 U/mL PK and 25-35 U/mL LD, final concentrations), 2 mM ATP, spectinomycin and streptomycin at various concentrations. The steady-state velocities were determined from the linear phase of the reaction progress curve and plotted as a function of the substrate concentration. Data were fit by nonlinear least squares methods with the Michaelis−Menten equation, *v*=(*V*_max_[*S*])/(*K*_m_+[*S*]), using GraphPad Prism 9 (GraphPad Software, Inc.) to calculate *K*_m_ and *k*_cat_ (**Supplementary Figure S3**). In this equation, *v* is the steady-state velocity; *V_max_* is the maximal velocity; [*S*] is the substrate concentration; *K_m_* is the Michaelis constant for the substrate; and the turnover rate *k*_cat_ is calculated from the equation *V*_max_ = *k*_cat_ [*E*], where [*E*] is the enzyme concentration.

### Quantitative RT−PCR analysis

To analyze the expression of the *aph(9)-Ic* and *aph(9)-Ic1*, overnight cultures of *S. maltophilia* 142 and 156 were diluted in fresh LB broth at a ratio of 1:100 and incubated to OD_600_ = 0.5, followed by the addition of 1/4 MIC spectinomycin (*S. maltophilia* 142, 4,096 μg/mL; *S. maltophilia* 156, 2,048 μg/mL) in the medium and further incubated for 1 h. Total RNA was extracted using RNAiso Plus (TaKaRa, Dalian, China) according to the manufacturer’s instructions. Total RNA was determined spectrophotometrically for RNA purity and concentration by an Implen NanoPhotometer (Implen GmbH, Munich, Germany). DNA-free RNA was confirmed by PCR amplification of the 16S rRNA gene of *S. maltophilia*. cDNA was synthesized using the Hiscript III All-in-one RT Super mix Perfect for qPCR Kit (Vazyme Biotech, Nanjing, China). RT Q-PCR was performed using SYBR qPCR Master Mix (Vazyme Biotech, Nanjing, China) in a CFX96TM Touch Real-Time PCR Detection System (Bio-Rad Laboratories, Hercules, CA, United States) to monitor the increase in fluorescence in real time. The primers used for quantitative RT−PCR (qRT−PCR) are listed in **Supplementary Table S2**. The relative expression of the genes was calculated by the 2^-ΔΔCT^ method (Livak and Schmittgen, 2001) using 16S rRNA as the internal control (Wu et al., 2019). Expression levels were compared with and without antibiotic treatment using one-way ANOVA (LSD test). P ≤ 0.05 was considered significant.

### Nucleotide sequence accession numbers

The chromosomal nucleotide sequences of the *S. maltophilia* strains 142 and 156 reported in this study have been deposited in GenBank under accession numbers CP098483 and JAMQEE000000000, respectively. The accession numbers for the novel resistance genes and their corresponding proteins are *aph(9)-Ic* (ON693243), *aph(9)-Ic1* (ON838098), APH(9)-Ic (USA18493.1), and APH(9)-Ic1 (MCM2524399.1).

## Results and discussion

### General features of the *S. maltophilia* strains 142 and 156 genomes

The *S. maltophilia* isolates 142 and 156 carrying the novel AG resistance genes designated *aph(9)-Ic* and *aph(9)-Ic1*, respectively, were collected from a tertiary hospital in Wenzhou, Zhejiang, China. Initially, the two isolates identified in a clinical laboratory using a MALDI-TOF mass spectrometer (BioMérieux, Marcy l’Etoile, France) were all *S. maltophilia*. The ANI results showed that the genomes of the isolates 142 and 156 had the highest identities of 98.36% and 97.22% with that of *S. maltophilia* NCTC10258 (NZ_LS483377.1) and *Pseudomonas hibiscicola* ATCC 19867 (GCF_000382065.1), respectively. *P. hibiscicola* is a heterotypic synonym of *S. maltophilia* (van den Mooter and Swings, 1990) in the genus *Stenotrophomonas* (Anzai et al., 2000). Currently, *P. hibiscicola* is not recognized by the National Center for Biotechnology Information (NCBI), so we named this isolate *S. maltophilia* 156. In addition, the sequence type of *S. maltophilia* 142 was ST84, while *S. maltophilia* 156 cannot be assigned into any known sequence type.

When analyzing the resistance phenotypes and the genotypes of the two isolates, according to the working draft genome sequences obtained from Illumina sequencing, we found that there was no corresponding function-characterized resistance gene annotated from the genomes, even though the two isolates showed very high MIC levels against spectinomycin. According to the resistance gene annotation, several function-characterized aminoglycoside-modifying enzyme genes were found in the genomes of the two isolates, such as one (*aph(3’)-IIc*) in *S. maltophilia* 142, and two [*aph(3’)-IIc* and *aac(6’)-Iz*] in *S. maltophilia* 156, but none of them intermediated resistance to spectinomycin (Li et al., 2003; Okazaki and Avison, 2007).

Instead, a putative APH(9)-I gene (finally designated *aph(9)-Ic* in this work) was annotated in each of the two genomes. To verify whether this predicted gene was functional, we cloned the potential resistance genes, and their resistance functions were further confirmed. To better understand the genetic background of the novel spectinomycin resistance determinant, the complete genome of *S. maltophilia* 142 was further finished. The whole genome of *S. maltophilia* 142 consisted of a chromosome (without a plasmid), which was 4,830,983 bp in length and encoded 4,378 ORFs with an average GC content of 66.20% (**Table 2** and **Supplementary Figure S4**). The number of contigs and the average contig length of the *S. maltophilia* 156 draft genome were 114 and 38,836 bp, respectively.

**Table 2:** General features of the *S. maltophilia* isolates 142 and 156 genomes.

	*S. Maltophilia* 142 (complete chromosome)	*S. maltophilia* 156 (working draft)
Size (bp)	4,830,983	4,427,356
GC content (%)	66.20	66.33
Predicted coding sequences (CDSs)	4,378	3,988
Proteins coding genes	4,350	3,945
Pseudo Genes	28	43
Protein coding (%)	99.36	98.92
Average ORF length (bp)	977	991
Average protein length (bp)	325	329
tRNAs	72	67
rRNA	4, 3, 3 (5S, 16S, 23S)	3, 3, 3 (5S, 16S, 23S)

### Resistance profiles of *S. maltophilia* 142 and *S. maltophilia* 156

The MICs of the tested antimicrobials for *S. maltophilia* strains 142 and 156 are shown in **Table 3**, where MICs are defined as the lowest concentration of an antimicrobial that will inhibit the visible growth of a microorganism after overnight incubation (Andrews, 2001). The *in vitro* susceptibility test showed that *S. maltophilia* 142 exhibited resistance to 6 (among those whose breakpoints were available from CLSI or US FDA) of the 23 antimicrobials tested, while *S. maltophilia* 156 showed resistance to 7 of them. *S. maltophilia* strains 142 and 156 also showed high MIC levels for spectinomycin (16,384 and 8,192 μg/mL, respectively), streptomycin (>128 μg/mL), ribostamycin (1,024 and 64 μg/mL, respectively), paromomycin (256 and 128 μg/mL, respectively), kanamycin (64 μg/mL), neomycin (>16 μg/mL), cefoxitin (>64 μg/mL), and fosfomycin (>64 μg/mL), which have no established breakpoints. *S. maltophilia* 142 was susceptible to many other antimicrobials, including tobramycin, ceftazidime, levofloxacin and chloramphenicol (**Table 3**).

**Table 3:** MIC values of various antimicrobials for seven bacterial strains (μg/mL).

Antimicrobial class	Antimicrobial	*S. maltophlia* 142	*S. maltophilia* 156	pMD19-T-*aph(9)-Ic*/DH5α	pMD19-T-*aph(9)-Ic1*/DH5α	ATCC25922	DH5α	pMD19-T/DH5α
Aminocyclitols	Spectinomycin	16,384	8,192	64	32	8	8	8
Aminoglycosides	Streptomycin	>128	>128	1	1	2	1	1
	Amikacin	128 (R)	64 (R)	1	1	1	1	1
	Ribostamycin	1,024	64	2	2	8	2	2
	Paromomycin	256	128	1	1	2	1	1
	Gentamicin	32 (R)	32 (R)	1	0.25	0.25	0.25	0.25
	Tobramycin	8 (S)	8 (S)	0.125	0.125	0.5	0.125	0.125
	Kanamycin	64	64	1	1	1	1	1
	Neomycin	>16	>16	1	1	1	1	1
	Sisomicin	2	4	0.25	0.125	0.25	0.125	0.25
β-Lactams	Ampicillin	32	128	>128	>128	4	2	512
	Cefoxitin	>64	>64	2	2	2	2	2
	Cefepime	32 (R)	64 (R)	2	0.25	0.125	0.125	0.125
	Ceftazidime	8 (S)	16 (R)	1	0.5	0.25	0.25	0.25
	Meropenem	64 (R)	64 (R)	0.25	0.03	0.03	0.03	0.03
	Aztreonam	>64 (R)	>64 (R)	0.25	0.125	0.125	0.125	0.125
Quinolones	Nalidixic acid	>4	>4	4	2	4	4	4
	Levofloxacin	1 (S)	1 (S)	0.03	0.03	0.03	0.03	0.03
Amphenicols	Chloramphenicol	8 (S)	8 (S)	4	8	4	8	8
Tetracyclines	Tetracycline	16	16	2	2	1	2	2
	Tigecycline	1	1	1	1	0.5	1	1
Phosphonic acid derivatives	Fosfomycin	>64	>64	2	2	2	2	2
Polymyxins	Colisitin B	>64 (R)	>64 (R)	2	1	1	1	1

### APH(9)-Ic confers resistance to spectinomycin

Compared with the control (*E. coli* DH5α harboring the vector pMD19-T only), the two recombinants with the cloned *aph(9)-Ic* variants (pMD19-T-*aph(9)-Ic*/*E. coli* DH5α and pMD19-T-*aph(9)-Ic1*/*E. coli* DH5α) increased the MIC levels of spectinomycin by 8- and 4-fold, respectively, while no significant increase in MIC level was identified for the other AGs (**Table 3**). Among the two recombinant clones, one from *S. maltophilia* 142 (*aph(9)-Ic*) showed a 2-fold greater increase in the MIC level of spectinomycin than that of the other one (*aph(9)-Ic1*).

### Kinetic parameters of APH(9)-Ic

The phosphotransferase activity and kinetic parameters of APH(9)-Ic were determined using purified APH(9)-Ic, and a variety of spectinomycin concentrations were used as substrates. The *k*_cat_ (1.09 ± 0.09) s^-1^, *K*_m_ (31 ± 5) μM, and the catalytic efficiency (*k*_cat_ */K*_m_ ratio) of (5.58 ± 0.31) × 10^4^ M^−1^·s^−1^ was observed for spectinomycin, while no significant catalytic efficiency was observed for streptomycin, which was consistent with the corresponding MIC level change of the recombinant strain (pMD19-T-*aph(9)-Ic*/DH5α) in the antimicrobial susceptibility test (**Table 3**). The gene encoding APH(9)-Ia was first identified from *Legionella pneumophila* in 1997 (Suter et al., 1997). Further study revealed that APH(9)-Ia effectively phosphorylates spectinomycin with *K*_m_ = 21.5 μM, *k*_cat_ = 24.2 s^-1^ and *k*_cat_ */K*_m_ of 1.1 × 10^6^ M^−1^·s^−1^ and did not bind to other AGs, including kanamycin, amikacin, neomycin, butirosin, streptomycin and apramycin (Thompson et al., 1998). APH(9)-Ic showed some common functional characteristics with APH(9)-Ia and APH(9)-Ib, such as the narrow substrate spectrum and regiospecificity of the enzyme (Lyutzkanova et al., 1997; Ramirez and Tolmasky, 2010).

### *aph(9)-Ic* and *aph(9)-Ic1* RNA expression after exposure to spectinomycin

**Figure 1:**
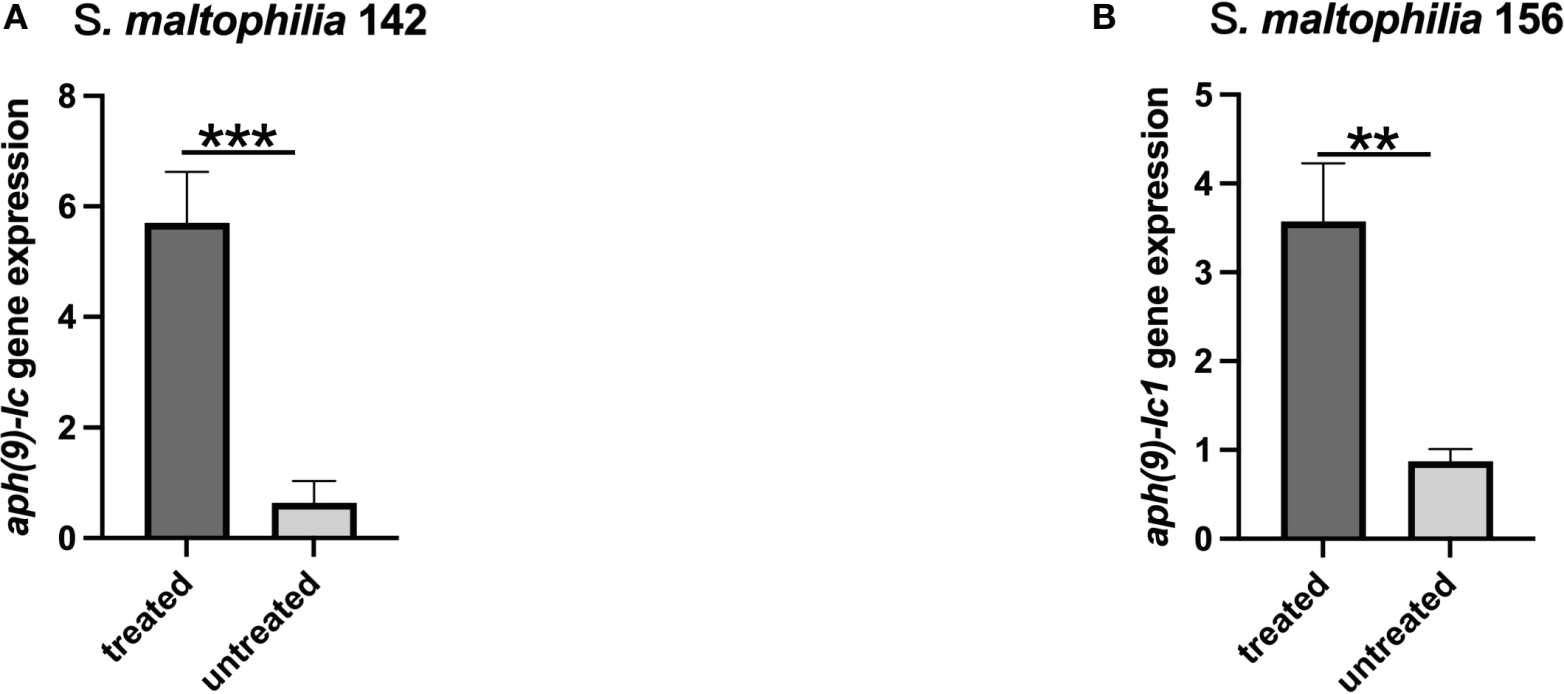
Expression levels of the *aph(9)-Ic* genes in *S. maltophilia* 142 and *S. maltophilia* 156 treated or untreated with spectinomycin. **(A)** *S. maltophilia* 142 treated with 4,096 μg/mL spectinomycin; **(B)** *S. maltophilia* 156 treated with 2,048 μg/mL spectinomycin. Bars represent the mean ± standard error, and all experiments were performed in triplicate. ** represents *P* < 0.01; *** represents *P* < 0.001.

To investigate the effect of spectinomycin on the expression of the *aph(9)-Ic* and *aph(9)-Ic1* genes, the mRNA levels of the two genes treated with or without spectinomycin were determined. The expression of the *aph(9)-Ic* and *aph(9)-Ic1* genes increased significantly (P < 0.01) after induction with 1/4 MIC spectinomycin for 1 h (**Figure 1**). Compared to the untreated control group, the expression of *aph(9)-Ic* in *S. maltophilia* 142 and *aph(9)-Ic1* in *S. maltophilia* 156 increased 8.95- and 4.08-fold, respectively.

### Comparative analysis of APH(9)-Ic

**Figure 2:**
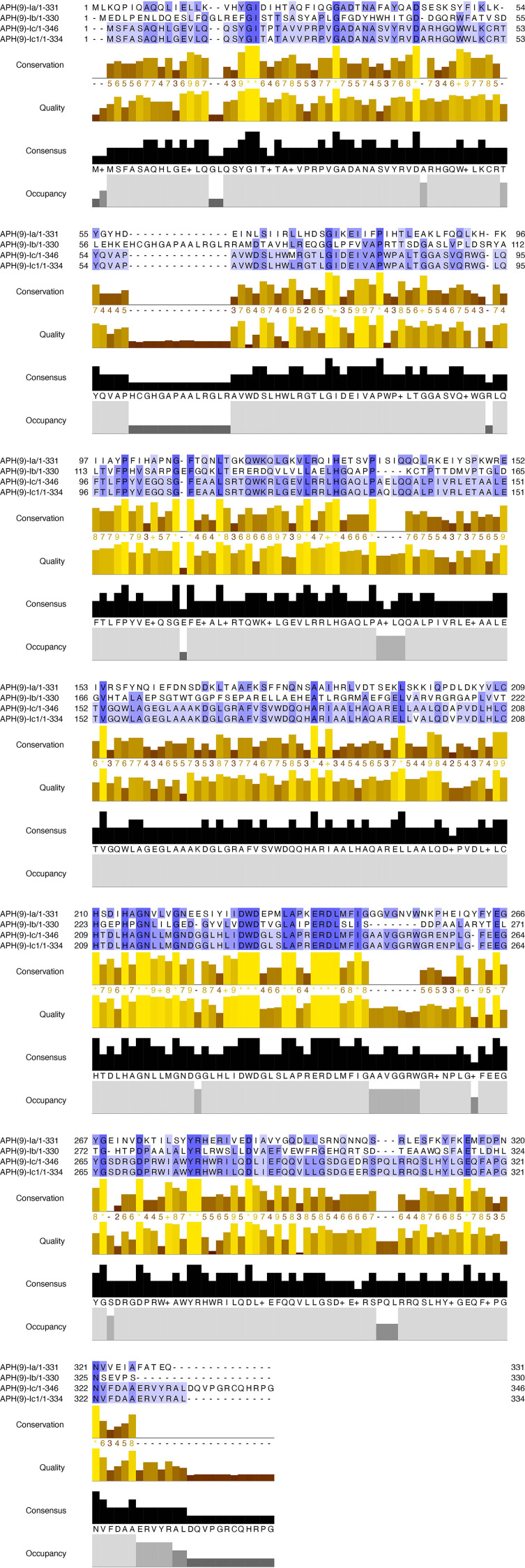
Multiple alignment of the amino acid sequences of APH(9)-Ic and other APH(9)-I subgroup proteins. The sequences and their accession numbers are: APH(9)-Ia (AAB58447.1), APH(9)-Ib (AAB66655.1), APH(9)-Ic (USA18493.1) and APH(9)-Ic1 (MCM2524399.1). Conservation, conservation of total alignment less than 25% gap; Quality, alignment based on Blosum62 scores; Consensus, percent of identity; Occupancy, number of aligned positions. The conserved motif sites are grayed. The figure was generated using Jalview (Waterhouse et al., 2009) with a ‘percent identity’ color scheme.

The nucleotide sequence length, number of encoded amino acids, predicted protein molecular mass and pI values of the *aph(9)-Ic* variants from *S. maltophilia* strains 142 and 156 are shown in **Table 4**. The protein from *S. maltophilia* 142 is composed of 346 amino acids, while the protein from *S. maltophilia* 156 consists of 334 amino acids which is 12 amino acids shorter than that of *S. maltophilia* 142. It turned out that the amino acid residues at positions 335-346 in APH(9)-Ic are absent in APH(9)-Ic1 (**Figure 2**). APH(9)-Ic1 (from *S. maltophilia* 156) shared 98.20% amino acid sequence identity with the corresponding amino acid sequence of APH(9)-Ic (from *S. maltophilia* 142). It could be concluded that the structural variation caused the MIC level difference between the two genes that the cloned *aph(9)-Ic* gene from *S. maltophilia* 142 showed a 2-fold MIC level to spectinomycin more than that of *aph(9)-Ic1* from *S. maltophilia* 156.

**Table 4:** Basic properties of *aph(9)-Ic* and *aph(9)-Ic1* and their proteins.

Gene name	Nucleotide sequence length (bp)	Number of encoded amino acids	Protein molecular mass (kDa)	Protein pI value (pH)
*aph(9)-Ic* (*S. maltophilia* 142)	1,041	346	38.43	6.57
*aph(9)-Ic1* (*S. maltophilia* 156)	1,005	334	37.12	5.61

**Figure 3:**
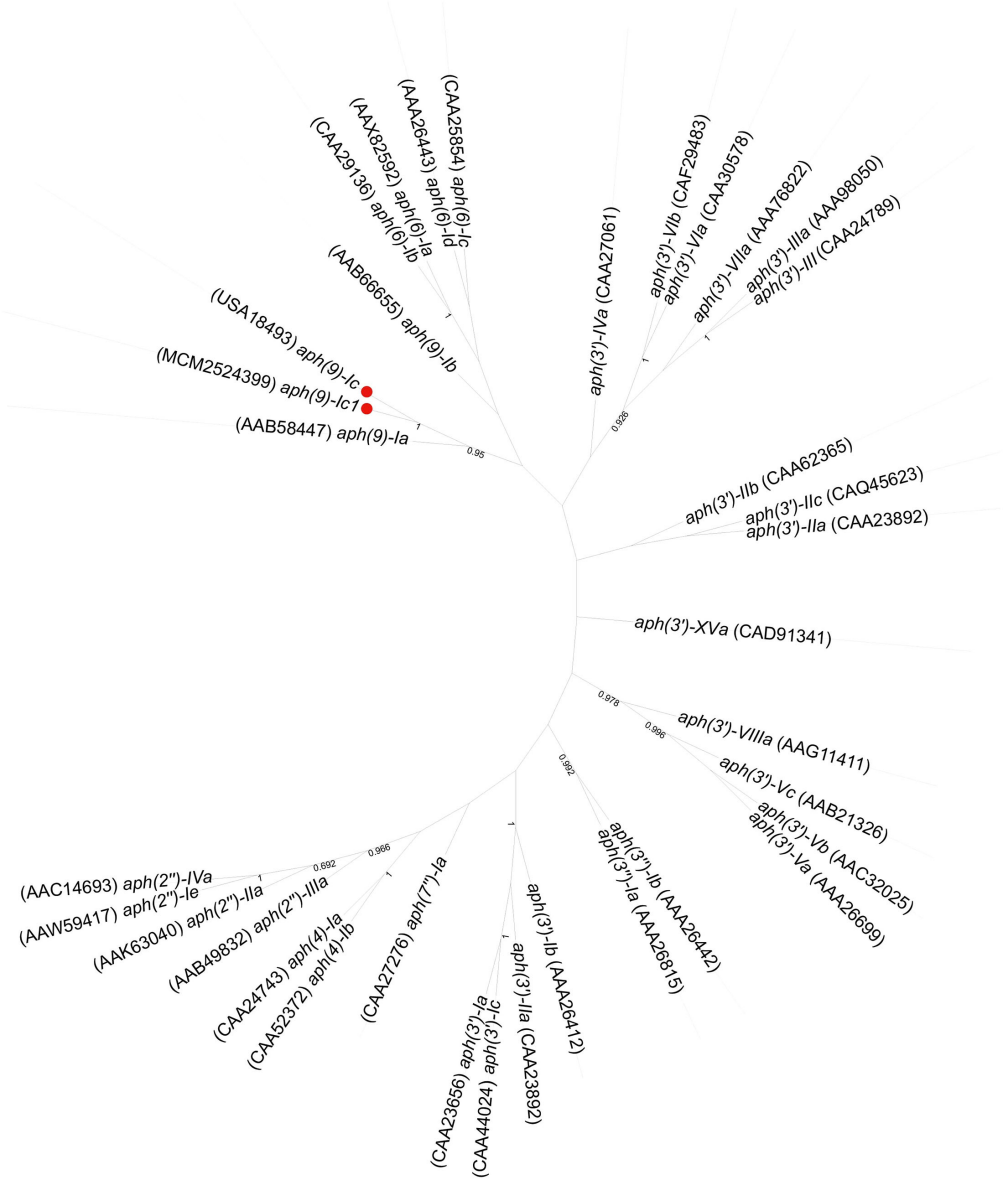
Phylogenetic tree showing the relationship of the APH(9)-Ic and APH(9)-Ic1 proteins with the other function-characterized APH genes. The proteins from this study are shown with a red dot.

A phylogenetic tree of APH(9)-Ic and the other function-characterized APH enzymes, including APH(3’), APH(3’’), APH(9), APH(7’), APH(2’’), APH(6) and APH(4), collected from the CARD (Mcarthur et al., 2013) and UniProtKB/Swiss-Prot (http://web.expasy.org/docs/swiss-prot_guideline.html) databases was constructed. The APH(9)-Ic proteins clustered closest to a branch composed of APH(9)-Ia (**Figure 3**). Among the function-characterized AG resistance proteins, APH(9)-Ic shared the highest amino acid sequence identities of 33.75% and 28.04% with APH(9)-Ia (AAB58447.1) and APH(9)-Ib (AAB66655.1), respectively, while APH(9)-Ic1 shared 34.06% and 28.69% identities with APH(9)-Ia (AAB58447.1) and APH(9)-Ib (AAB66655.1), respectively. This finding indicates that the proteins identified in this work are novel AG *O*-phosphotransferases of the APH(9)-I family (**Figure 3**). According to the nomenclature criteria recommended by Shaw (Shaw et al., 1993), the novel AG resistance protein from *S. maltophilia* 142 was thus designated APH(9)-Ic.

### Genetic context of the *aph(9)-Ic* gene

**Figure 4:**
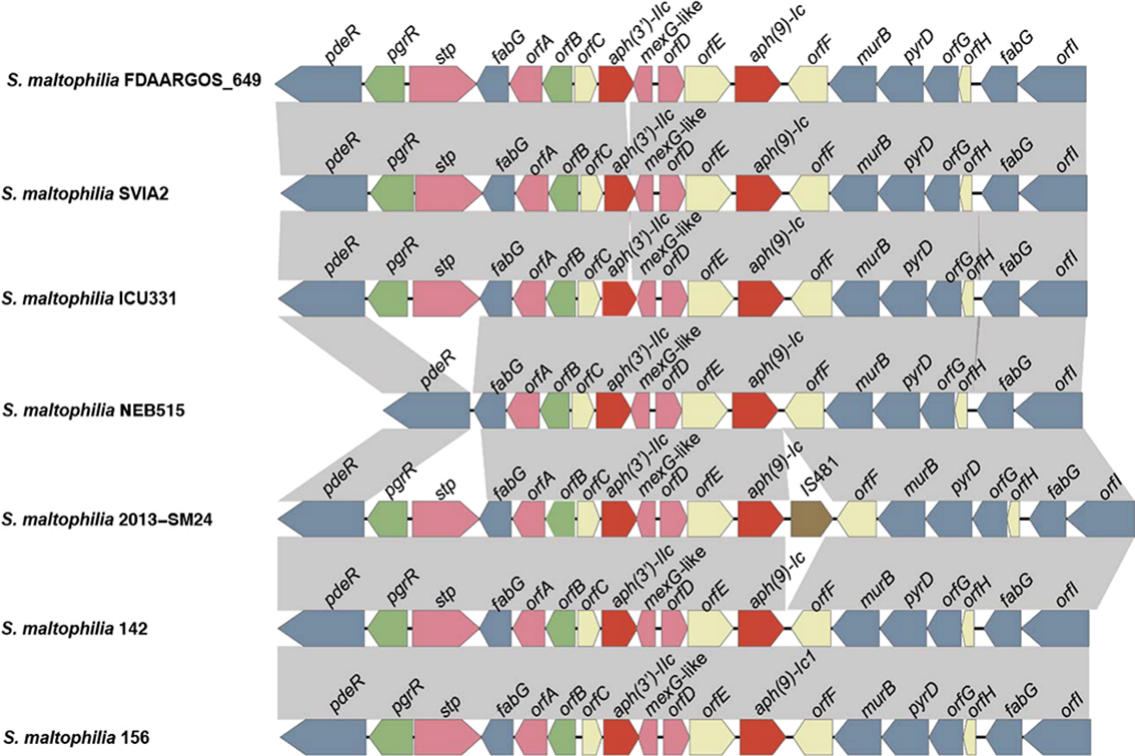
Comparative analysis of the genomic context of the *aph* gene-related regions. ORFs are shown as arrows drawn to scale to indicate the direction of transcription and colored based on gene function classification. Gray shading denotes regions of homology. From left to right, these genes and functions are as follows: *pdeR*, cyclic di-GMP phosphodiesterase; *pgrR*, helix-turn-helix (HTH)-type transcriptional regulator; *stp*, multidrug resistance protein; *fabG*, 3-oxoacyl-[acyl-carrier-protein] reductase; *orfA*, the Gcn5-related N-acetyltransferases (GNAT) family; *orfB*, ArsR family transcriptional regulatory protein; *orfC*, transmembrane protein; *aph(3’)-IIc*, aminoglycoside 3’-phosphotransferase; *mexG*-like, multidrug efflux RND transporter inhibitory subunit; *orfD*, tetracycline repressor protein family; *orfE*, transmembrane protein; *aph(9)-Ic*, aminoglycoside 9-phosphotransferase; *orfF*, transmembrane protein; *murB*, UDP-N-acetylenolpyruvoylglucosamine reductase; *pyrD*, dihydroorotate dehydrogenase; *orfG*, methyltransferase; *orfH*, transmembrane protein; *fabG*, 3-oxoacyl-[acyl-carrier-protein] reductase; *orfI*, dehydrogenase.

To explore the genetic environment of *aph(9)-Ic*, the structures of the approximately 10-kb flanking regions of *aph(9)-Ic* were analyzed and are shown in **Figure 4**. Comparative analysis of the *aph(9)-Ic* gene-flanking regions of *S. maltophilia* strains 142 and 156 with the *aph(9)-Ic* gene*-*carrying fragments from other strains revealed that the regions adjacent to *aph(9)-Ic* were conserved in the corresponding parts of the chromosomal sequences of the *S. maltophilia* strains, with the exception that an IS*481* was present next to *aph(9)-Ic* in the sequence from *S. maltophilia* 2013-SM24. Notably, several antimicrobial resistance-related genes, such as *stp*, *orfA*, *mex-G* like, *orfD* and *aph(3’)-IIc*, were present in the upstream regions of the *aph(9)-Ic* genes. The presence of *aph(3’)-IIc* may confer bacterial resistance to paromomycin, kanamycin and neomycin (**Table 3**) (Okazaki and Avison, 2007). Due to the high degree of conservation of the genetic context of *aph(9)-Ic* and related genes in species of the genus *Stenotrophomonas*, *aph(9)-Ic* may be an intrinsic gene that is useful for resistance against spectinomycin produced naturally by *Streptomyces* (Gomes and Menawat, 1998) or introduced artificially.

## Conclusion

In this work, we identified a novel chromosomally encoded AG 9-*O*-phosphotransferase, designated APH(9)-Ic. The new AG resistane enzyme exhibits high amino acid sequence identities (28.04-33.75%) with all function-characterized APH(9)-I subgroup proteins of APH(9)-Ib and APH(9)-Ia and confers resistance to spectinomycin with an efficient catalytic activity [*k*_cat_/*K*_m_ ratio (5.58 ± 0.31) × 10^4^ M^−1^·s^−1^]. The identification and characterization of novel resistance genes in an uncommon opportunistic pathogen will provide evidence of the increasing number of resistance mechanisms in microbial populations and the clinic to effectively treat the infectious diseases caused by the bacteria.

## Data availability statement

The datasets presented in this study can be found in online repositories. The names of the repository/repositories and accession number(s) can be found in the article/**Supplementary Material**.

## Ethics statement

Individual patient data were not involved, and only anonymous clinical residual samples during routine hospital laboratory procedures were used in this study. This study was approved by the ethics committee of the Second Affiliated Hospital and Yuying Children’s Hospital of Wenzhou Medical University, Wenzhou, Zhejiang, China.

## Author contributions

Conceived and designed the experiments: KL, HZ, YH, GW, QB and WJ. Performed the experiments: WS, JL, MG, AL, SL, LZ, XZ and QL. Data analysis and interpretation: WS, CF, HL, XL, GW, QB and WJ. Drafting of the manuscript: WS, JL, QB and WJ. All authors contributed to the article and approved the submitted version.

## Supplementary material

Supplementary Figure 1 Multiple sequence alignment for generating the phylogenetic tree. The figure was generated using Jalview (Waterhouse et al., 2009) with a ‘percent identity’ color scheme.

Supplementary Figure 2 SDS−PAGE gel of the recombinant protein. A, His6-tagged APH(9)-Ic. Lane M, protein maker in kDa; lane 1, flow-through solution; lanes 2-6, washing solution; lanes 7-12, 50 mM imidazole eluate. B, APH(9)-Ic free of His6-tag. lane M, protein maker in kDa; lane 1, His6-tagged APH(9)-Ic; lanes 2-3, APH(9)-Ic free of His6-tag.

Supplementary Figure 3 Three repeats of Michaelis−Menten equation fitting.

Supplementary Figure 4 Genome map of the chromosomal sequence of *S. maltophilia* 142. From outside to inside: circles 1 and 2 display predicted ORFs encoded in the forward and reverse strands, and circles 3, 4 and 5 represent the GC content, GC skew, and scale in kb of the *S. maltophilia* 142 genome, respectively.
