# Supplementary material for: Functional characterization of a novel aminoglycoside phosphotransferase, APH(9)-Ic, and its variant from Stenotrophomonas maltophilia
Source: Front Cell Infect Microbiol. 2023 Jan 9;12:1097561. doi: 10.3389/fcimb.2022.1097561 (PMC9868417; doi:10.3389/fcimb.2022.1097561)
Supplement: Supplementary file 1 [file Table_1.docx]

**TABLE S1 | Cloning primers for the *aph(9)-Ic* and *aph(9)-Ic1* genes.**

| Primer^a^ | Sequence (5’–3’)^b^ | Restriction endonuclease | Vector | Annealing  temperature (˚C) | Amplicon size (bp) |
| --- | --- | --- | --- | --- | --- |
| 142pro-*aph(9)-Ic*-F | AGTGTCATCGCCGCCTGC |  | pMD19-T | 65 | 1,984 |
| 142pro-*aph(9)-Ic*-R | GGCCCTGTTCGTCTGGGTCT |  | pMD19-T |  |  |
| 156pro-*aph(9)-Ic1*-F | TCGTGGCAGTTCGCACGC |  | pMD19-T | 60 | 1,197 |
| 156pro-*aph(9)-Ic1*-R | CTACTCGGGGCGATGCTG |  | pMD19-T |  |  |
| 142orf-*aph(9)-Ic*-F | CGCGGATCCGACGACGACGACAAGATGTCATTCGCTTCGGCGCAGCTTCGCTTCGGCGCAGC | *Bam*HⅠ+ Enterokinase | pCold I | 65 | 1,118 |
| 142orf-*aph(9)-Ic*-R | CCAAGCTTGCTGCGCGCGATAGTCGACT | *Hind*Ⅲ | pCold I |  |  |

^a^ primers with “orf” were used to clone the ORF of the *aph(9)-Ic* gene, and primers with “pro” were used to clone the *aph(9)-Ic* and *aph(9)-Ic1*genes with their promoter regions.

^b^ The underlined sequences represent the restriction endonuclease sites.
