# Supplementary material for: Functional characterization of a novel aminoglycoside phosphotransferase, APH(9)-Ic, and its variant from Stenotrophomonas maltophilia
Source: Front Cell Infect Microbiol. 2023 Jan 9;12:1097561. doi: 10.3389/fcimb.2022.1097561 (PMC9868417; doi:10.3389/fcimb.2022.1097561)
Supplement: Supplementary file 2 [file Table_2.docx]

**TABLE S2 | Primers for Q-PCR.**

| Primer^a^ | Sequence (5’–3’) | Annealing temperature (◦C) | Amplicon size (bp) |
| --- | --- | --- | --- |
| q-*aph(9)-Ic*-F | CAGTCACTGCACTACCTGGG | 60 | 106 |
| q-*aph(9)-Ic*-R | GATGCTGGCAACGGCCT |  |  |
| q-*aph(9)-Ic1*-F  q-*aph(9)-Ic1*-R | GGACCTGATCGAGTTCCAGC  GCCCAGATAGTGCAACGACT | 60 | 91 |
| 16S-F | GACCTTGCGCGATTGAATG | 60 | 75 |
| 16S-R | CGGATCGTCGCCTTGGT |  |  |

^a^ primers with “q” were used for the *aph(9)-Ic* and *aph(9)-Ic1* genes, and primers with “16S” was used for the 16S RNA gene.
