# Supplementary figures and images for: Functional characterization of a novel aminoglycoside phosphotransferase, APH(9)-Ic, and its variant from Stenotrophomonas maltophilia
Source: Front Cell Infect Microbiol. 2023 Jan 9;12:1097561. doi: 10.3389/fcimb.2022.1097561 (PMC9868417; doi:10.3389/fcimb.2022.1097561)

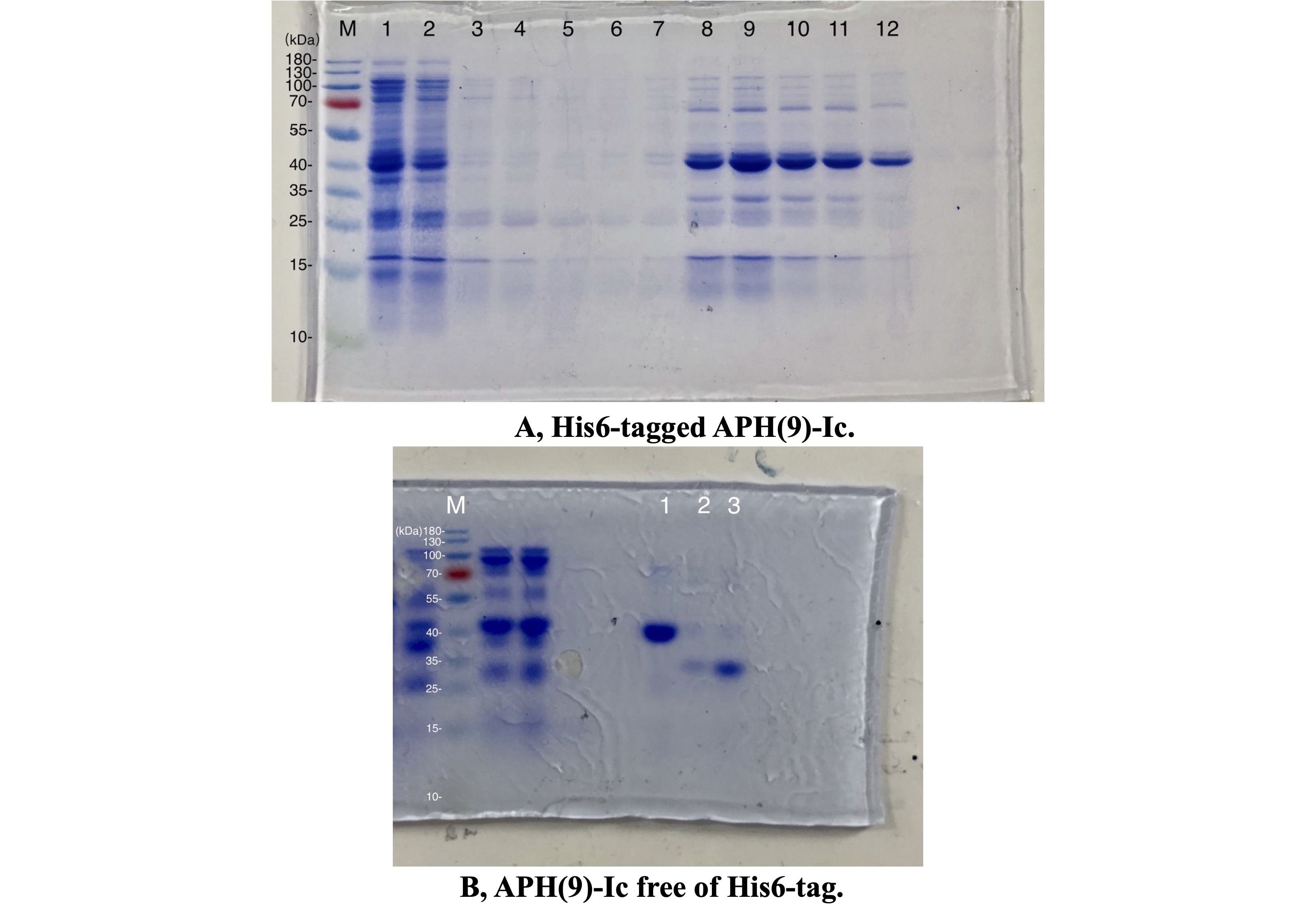

Supplement: Supplementary Figure 2 — SDS−PAGE gel of the recombinant protein. A, His6-tagged APH(9)-Ic. Lane M, protein maker in kDa; lane 1, flow-through solution; lanes 2-6, washing solution; lanes 7-12, 50 mM imidazole eluate. B, APH(9)-Ic free of His6-tag. lane M, protein maker in kDa; lane 1, His6-tagged APH(9)-Ic; lanes 2-3, APH(9)-Ic free of His6-tag. [file Image_2.jpeg]

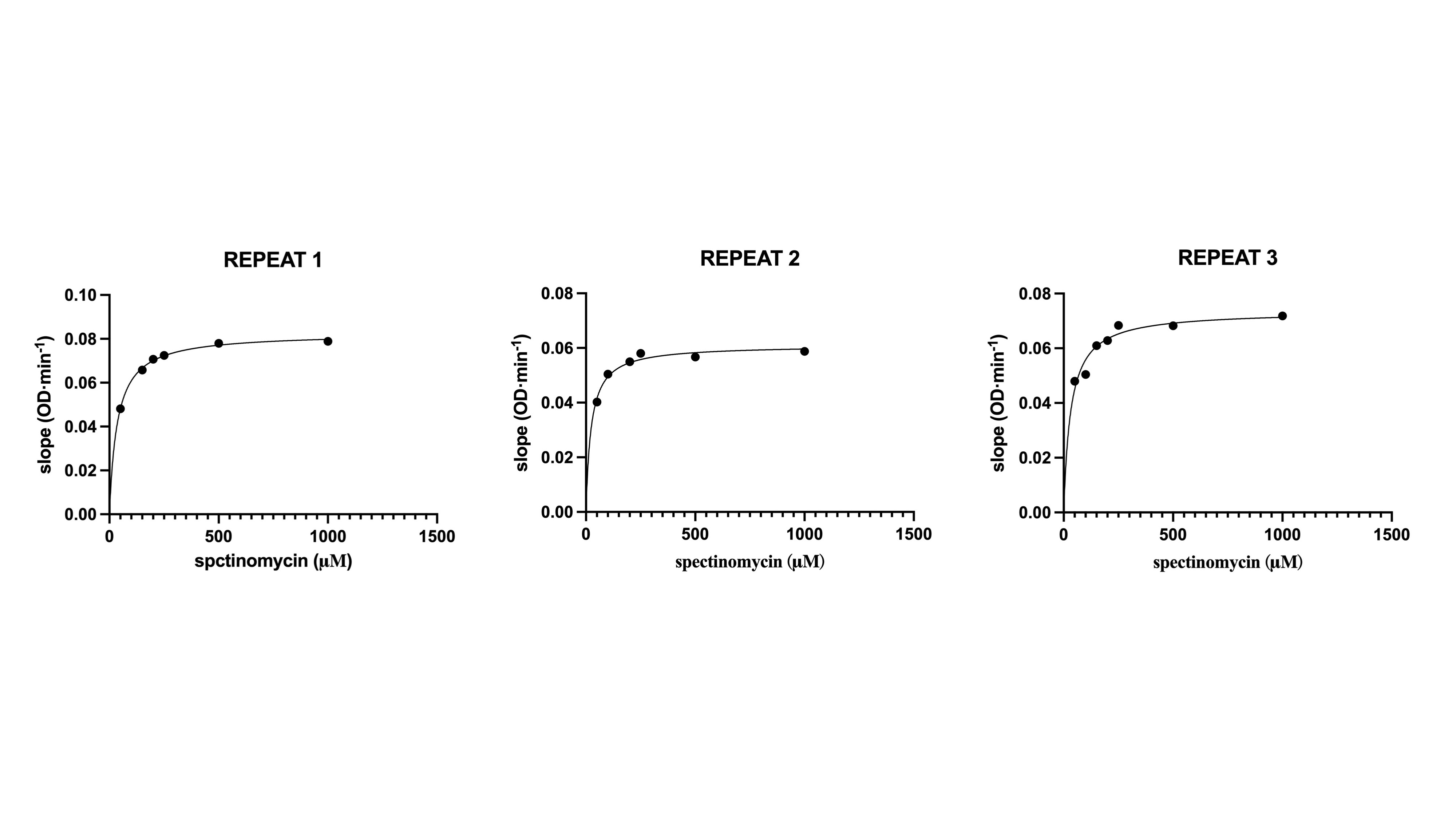

Supplement: Supplementary Figure 3 — Three repeats of Michaelis−Menten equation fitting. [file Image_3.jpeg]

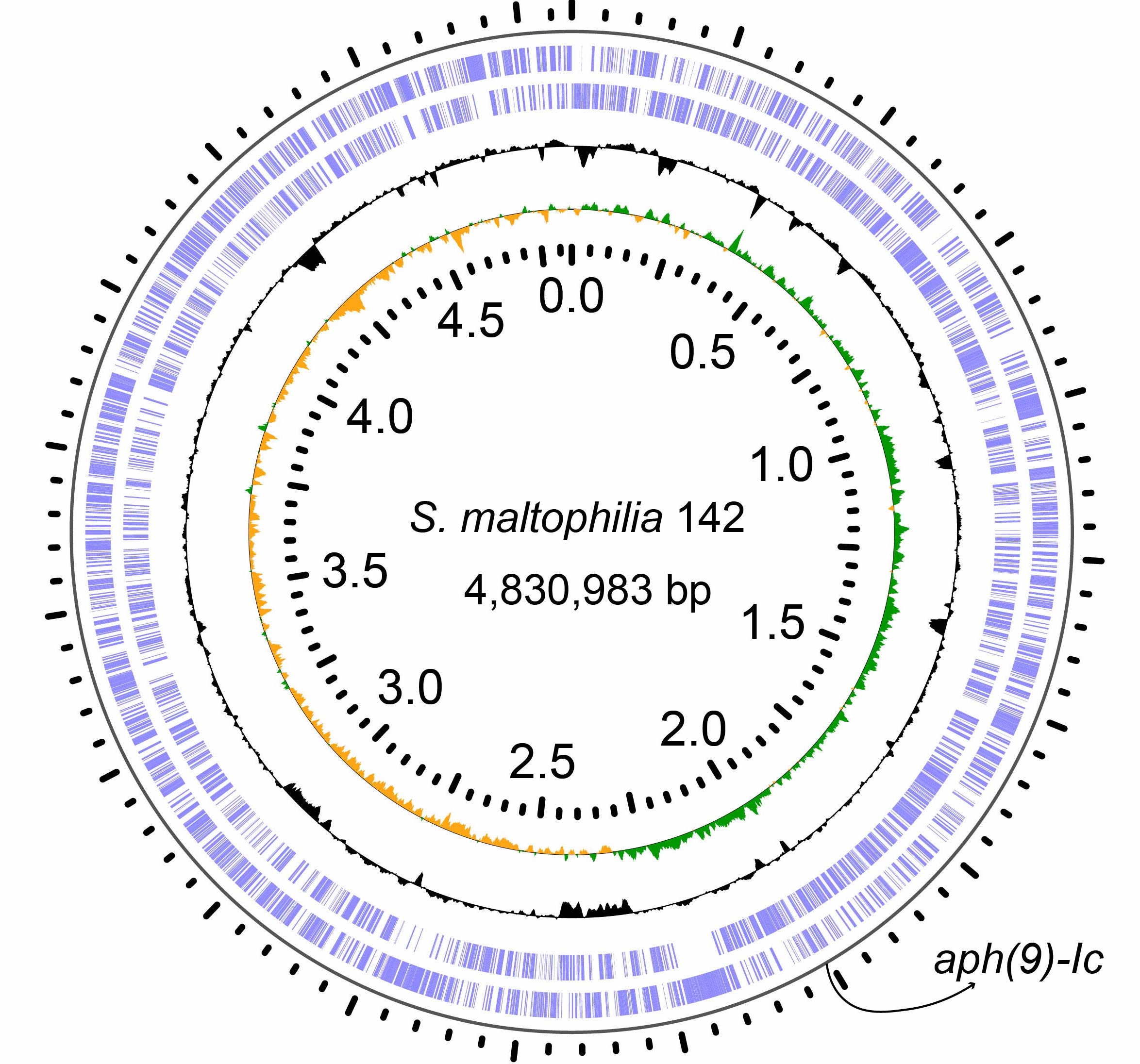

Supplement: Supplementary Figure 4 — Genome map of the chromosomal sequence of S. maltophilia 142. From outside to inside: circles 1 and 2 display predicted ORFs encoded in the forward and reverse strands, and circles 3, 4 and 5 represent the GC content, GC skew, and scale in kb of the S. maltophilia 142 genome, respectively. [file Image_4.jpeg]
